# A Study on Acute Myocardial Infarction and Its Prognostic Predictors

**DOI:** 10.7759/cureus.34775

**Published:** 2023-02-08

**Authors:** Manduri Sathvik, Eswar Chand Satyendra Sai Kalva, Gonji Suma

**Affiliations:** 1 Department of Internal Medicine, NRI Medical College and General Hospital, Guntur, IND; 2 Department of Community Medicine, Katuri Medical College and Hospital, Guntur, IND

**Keywords:** nstemi, stemi, prognosis, serum uric acid, myocardial infarction, killip’s class

## Abstract

Introduction

Acute Myocardial Infarction (AMI) is a serious cardiac event characterized by the sudden death of heart muscle tissue due to the obstruction of blood flow to the heart. It is a leading cause of death and disability worldwide. The relationship between AMI and serum uric acid levels is an area of ongoing research. Serum uric acid is a byproduct of purine metabolism and is typically present in the blood at low levels. Elevated levels of uric acid have been linked to several cardiovascular risk factors, including hypertension, diabetes, and hyperlipidemia. This has led to the investigation of the relationship between uric acid levels and AMI.

Materials and Methods

In this study, 100 individuals who were presented with acute myocardial infarction were included. All patients were categorized into four Killip’s classes based on history, clinical examination, and lab investigation. Subsequently, the four Killip’s classes were co-related with the serum uric acid of the patient.

Results

Serum uric acid levels were high in males compared to females. serum uric acid levels were high in Killip’s class III (7.24) and IV (7.57) compared to class I (4.48) and II (5.26). There was no significant correlation between serum uric acid and the co-morbidities like diabetes and hypertension, with a p-value of 0.48.

Conclusion

An increase in Killip Class is positively correlated with an increase in blood uric acid levels. Uric acid can therefore be utilized as a prognostic indicator in individuals who present with myocardial infarction.

## Introduction

Non-communicable diseases are becoming more significant since they have displaced infectious diseases as the leading cause of disability, morbidity, and premature mortality, demonstrating the epidemiological shift [1]. In India, cardiovascular disease (CVD) is a silent epidemic, and the prevalence of heart diseases has increased fourfold during the past 40 years. The number of CVD patients increased dramatically from 271 million in 1990 to 523 million in 2019, as did mortality, which increased from 12.1 million in 1990 to 18.6 million in 2019 [2]. Rapid urbanization and lifestyle modifications such as inactivity, an unwholesome diet, obesity, dyslipidemia, smoking, increased blood pressure, and diabetes has increased the prevalence of coronary heart disease during the past 20 years [3,4]. In conclusion, acute coronary syndrome (ACS) is a kind of CVD, and cardiovascular disease (CVD) is an umbrella term that covers a variety of illnesses that affect the heart and blood vessels.

The term “acute coronary syndrome" (ACS) refers to a variety of conditions. Acute Myocardial Infarction (AMI) can present as Non-ST Elevation Myocardial Infarction (NSTEMI), ST Elevation MI (STEMI), or unstable angina (UA) [5]. Notably, all of these illnesses may have a similar clinical appearance and symptoms. In ACS, relieving or limiting ischemia, preventing reinfarction, and improving outcome and well-being are the main therapy objectives. AMI risk stratification is carried out using several clinical evaluations and scores; Killip’s classification is one of them [6]. Killip classified AMI patients into four groups to detect the severity of left ventricular dysfunction and predict mortality. 

Class I: No signs of LV failure

Class II: Presence of S3 heart sound on auscultation and basal crepitation

Class III: Pulmonary edema

Class IV: Cardiogenic shock

The predicted mortality rates for the four classes are 6%, 17%, 38%, and 81%, respectively [6].

A significant risk factor and reliable predictor of cardiovascular illnesses is arterial stiffness [7]. Age and other medical conditions exacerbate arterial stiffness, which indicates vascular flexibility and function. According to some studies, uric acid can cause vascular endothelial dysfunction by inducing oxidative stress and inhibiting endothelial nitric oxide synthase, as well as by encouraging the growth of vascular smooth muscle cells and amplifying the vasoconstrictive effects of angiotensin II, endothelin, and thromboxane, which lead to subclinical changes in arterial structure [8]. Additionally, there is a strong relationship between uric acid and markers of arterial stiffness. Uric acid is a significant risk factor and predictor of cardiovascular events, such as acute myocardial infarction, atherosclerosis, and stroke [7,9]. With this knowledge, this study aims to investigate the association between Killip's class and serum uric acid levels in acute myocardial infarction.

## Materials and methods

A descriptive study was conducted in a tertiary care hospital in Guntur from April 2021 to September 2022 among 100 patients who were admitted to the emergency room with resting chest pain lasting more than 30 minutes after gaining ethics committee approval. Patients or their families were asked for their informed consent. Until a sample size of 100 was reached, a convenience sampling technique was utilized.

Inclusion and exclusion criteria

Patients with STEMI or NSTEMI with resting chest pain lasting more than 30 minutes, new ST/T changes or new left bundle branch block, or presence of pathological Q waves on ECG or ECHO showing regional wall motion abnormality and raised cardiac enzymes (CK-MB, troponins) more than 99^th^ percentile for the upper reference value, were all included in the study. Patients with other illnesses and drugs which are known to raise SUA levels were excluded from the study.

A semi-structured questionnaire was used to collect patients' socio-demographic details, presenting complaints, risk factors, and other pertinent clinical data. Blood pressure, random blood sugar, ECG or ECHO, chest X-ray, Killip's classification, and COBRA INTEGRA/COBAS C SYSTEM, which uses the uricase method to determine the quantity of uric acid in serum, were utilized as study tools. According to a study by Kuwabara M., the reference range for uric acid was 3 to 5 mg/dl in males and 2 to 4 mg/dl in females. Clinical examination, auscultatory findings for class II, chest X-ray findings for class III, and vital signs, clinical condition, and ECG of the patient for class IV were used to classify patients into Killip's classes. The pertinent data is subsequently entered into a Microsoft Excel master chart (Redmond, USA) and statistically assessed by IBM Corp. Released 2011. IBM SPSS Statistics for Windows, Version 20.0. Armonk, NY: IBM Corp.

## Results

The 100 patients in the current study had either STEMI or NSTEMI, and their ages ranged from 53.76 ± 8.056. Sixty-five percent of the study subjects were males, and out of them, 52 were smokers. Additionally, a large majority of persons, 66% and 72% of them, respectively, have a history of hypertension and diabetes mellitus. The distribution of sociodemographic profiles and risk factors were shown in Table 1.

**Table 1:** Distribution of socio-demographic profile, and risk factors among study subjects (n=100).

Variables	Percentage (%)
Age in years	
35-39	8.0
40-44	6.0
45-49	14.0
50-54	28.0
55-59	18.0
60-64	17.0
>65	9.0
Gender	
Male	65.0
Female	35.0
History of smoking	
Non- smokers	47.0
Smokers	53.0
Distribution of diabetes	
Non- diabetics	34.0
Diabetics	66.0
Distribution of hypertension	
Non- hypertensives	30.0
Hypertensives	70.0

Out of the 100 individuals, 47% had anterior wall MI, 12% had anterior wall MI and lateral wall MI, 34% had inferior wall MI, and 7% had non-ST elevation MI (Table 2).

**Table 2:** Distribution of types of AMI in the study population (n=100). AWMI: Anterior wall MI, LWMI: Lateral wall MI, IWMI: Inferior wall MI, NSTEMI: Non-ST elevation MI

Region of the AMI	Percentage (%)
AWMI	47.0
AWMI/LWMI	12.0
IWMI	34.0
NSTEMI	7.0
Total	100.0

**Figure 1:**
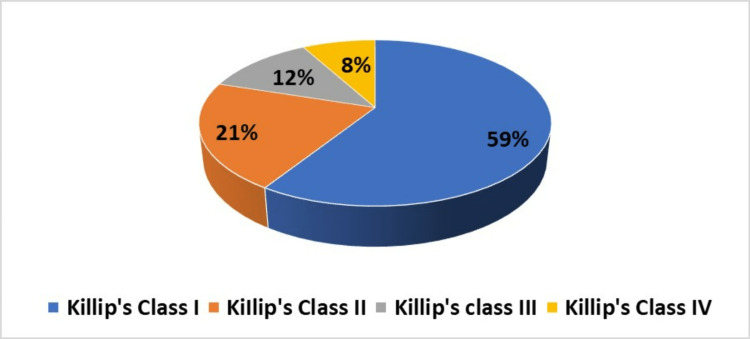
Distribution of study population based on KILLIP’S class.

KILLIP classes I and II made up around 80% of the 100 patients in our study, whereas classes III and IV (20%) (Figure 1).

**Figure 2:**
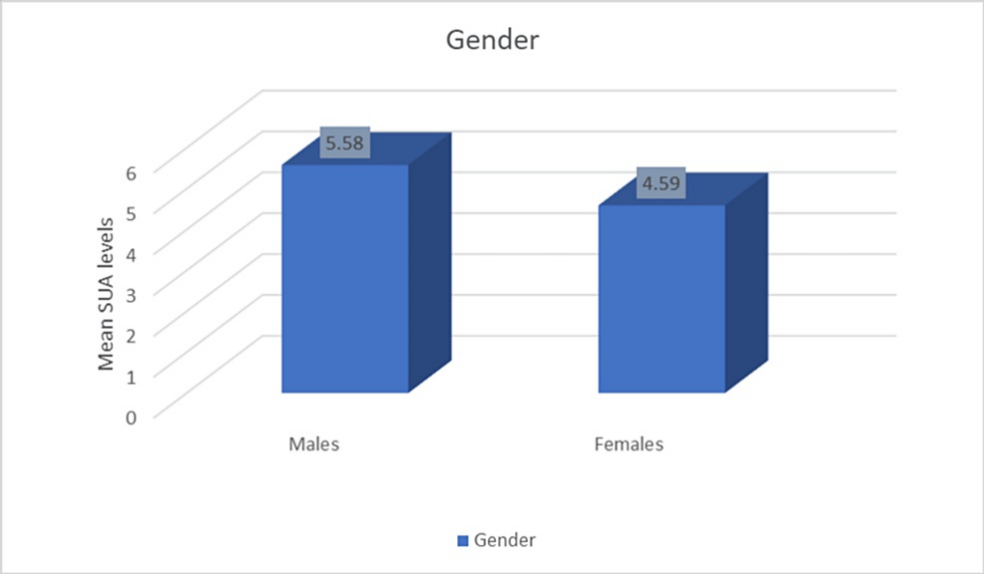
Distribution of mean uric acid levels among males and females.

Males have higher mean uric acid levels compared to females (Figure 2).

**Figure 3:**
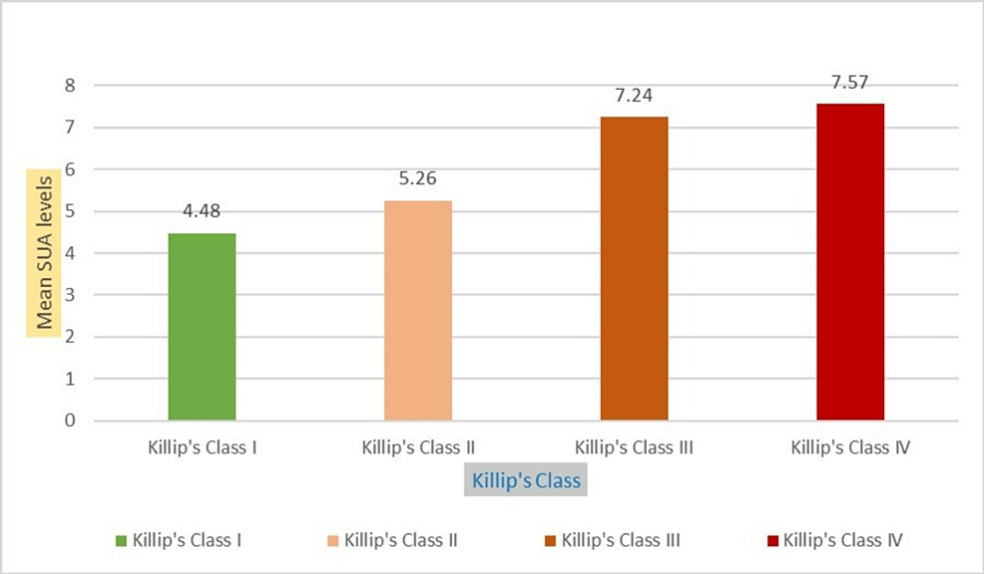
Relationship between SUA levels and Killip class.

In the present study, SUA levels were substantially higher in class IV and class III patients. By using the Kruskal-Wallis test, the mean variance was statistically significant (p=0.000), as shown in Table 3 and Figure 3.

**Table 3:** Relationship between SUA levels and Killip class.

Killip class	SUA Mean	Mean Rank	P
I	4.48	25.05	0.000
II	5.26	38.32	
III	7.24	62.05	
IV	7.57	64.32	

By using the Kruskal-Wallis test, it was shown that there was no statistically significant correlation between mean SUA levels and the presence of diabetes or hypertension (p=0.484) (Table 4).

**Table 4:** Association between SUA and hypertension and/or diabetics.

Adjustable	Mean SUA	SD	P-value
Have- hypertension and diabetes	5.545	1.547	0.484
Either diabetes or hypertension seen	5.029	1.3445	
Don’t have hypertension/diabetes	4.781	.4163	
Total	5.223	1.418	

## Discussion

In this study, blood uric acid levels in AMI were measured and correlated with Killip class among 100 study subjects. Our investigation showed that SUA levels are higher in Killip’s classes III and IV, with a mean of 7.57 and 7.24, respectively. Similarly, a study by Nadkar MY et al. [10] revealed that SUA levels were higher in classes III and IV compared to classes I and II. Another study by Bos MJ et al. [9] showed that SUA is the strongest risk factor for AMI. To contradict these studies, a study by Chen L et al. [11] stated that SUA levels were positively correlated with serum triglyceride levels but not with the severity of coronary artery disease. Whereas a study by Xue T et al. [12] revealed that only a stable high level of SUA was associated with an increased risk of MI, while a change in SUA in any direction was not associated with the risk of MI. A study by Kojima S et al. [13] stated that the combination of SUA and Killip’s class is a good predictor of mortality in patients with MI. A study conducted by Maryam M et al. [14] showed that patients with heart failure (the cases group), as compared to the control group, demonstrated a noticeably higher amount of uric acid. Additionally, individuals with STEMI had uric acid levels that were significantly greater than those with heart failure who did not have a STEMI. A study by Tsai TH [15] showed that even in patients undergoing primary percutaneous coronary intervention, Killip III continues to be a highly and independently reliable predictor of 30-day and one-year death in ST-segment elevation myocardial infarction patients.

According to our study and also stated by Taniguchi Y et al. [16], there was no significant relationship between hypertension and diabetes and SUA levels in our study. [16]. To contradict this, many studies proved a positive relationship between diabetes and SUA [17,18]. However, it was unclear why earlier research discovered a positive relationship between uric acid and diabetes.

Limitations

To find out the results of the interaction between SUA and Killip's class, we did not follow up with the patients. We conducted our study with 100 participants; a larger sample size could produce more conclusive results.

## Conclusions

Based on the above findings, we conclude that the SUA levels are directly proportional to Killip’s class. Therefore, the risk of mortality due to AMI increases as uric acid levels rise. So, we can use serum uric acid levels as a predictor of cardiovascular disease.
